# Retrieval-Augmented Generation Versus GPT-4o for Patient-Facing Gynecological Cancer Information: Quality Evaluation

**DOI:** 10.2196/90139

**Published:** 2026-04-24

**Authors:** Stephen Pearson, Mimi Reyburn, Conor Foley, Alison Finch, Suzanne Bench, Timothy Bonnici, Louise Rose

**Affiliations:** 1 Critical Care, University College London Hospitals NHS Foundation Trust, London, England, United Kingdom; 2 University College London Hospitals NHS Foundation Trust, London, United Kingdom; 3 Anaesthetic Department, Whittington Health NHS Trust, London, England, United Kingdom; 4 School of Nursing and Midwifery, London South Bank University, London, England, United Kingdom; 5 Florence Nightingale Faculty of Nursing, Midwifery and Palliative Care, King's College London, King's College London, Strand, London, England, WC2R2LS, United Kingdom, 44 02078365454

**Keywords:** gynecological neoplasms, patient education as topic, health literacy, large language models, retrieval-augmented generation, readability, GPT-4o

## Abstract

Retrieval-augmented generation improved overall quality scores for patient-facing gynecological cancer information mainly through better source attribution.

## Introduction

Health literacy, which is the ability to obtain, process and understand basic health information, varies widely. Furthermore, situational stress influences the ability to comprehend health information [1]. High-quality accessible information underpins shared decision-making. Yet people with cancer frequently report information overload and unmet needs at diagnosis and during treatment [2]. Producing timely, personalized, and readable health-related materials is human-resource intensive, yields static documents, and can result in duplicated effort and variable quality [3]. Large language models (LLMs) may offer a solution through rapid generation and adaptation of text. However, LLMs may hallucinate, cite unverifiable sources, and produce materials misaligned with literacy needs [4]. Retrieval augmented generation (RAG) may address these issues as it grounds LLM outputs in verified information as opposed to proprietary LLMs that rely on training data alone [5].

Our objective was to compare base GPT-40 with GPT-4o enhanced with a tailored gynecological cancer knowledgebase (RAG GPT-4o) in order to determine whether RAG improves the quality of AI-generated patient-facing information.

## Methods

### Study Design

We compared two GPT-4o configurations to answer frequently asked gynecological cancer questions compiled from patient queries and service information resources. For the RAG GPT-4o intervention arm, a retrieval layer supplied passages from a curated UK knowledge base restricted to Macmillan Cancer Support [6] and The Eve Appeal [7] resources. The Base GPT-4o control arm generated answers without retrieval of external sources. RAG GPT-4o ran via an application programming interface (API); Base GPT-4o ran via the web interface.

We compiled 17 questions and generated two responses per question (Base GPT-4o via ChatGPT web interface; RAG GPT-4o via API), producing 34 outputs. Prompts were held constant across configurations. Paired model outputs for each question formed the unit of comparison. Seven expert raters, each, evaluated eight paired outputs, while an LLM-judge evaluated all 17 paired outputs. Application Programming Interface generation settings were recorded for reproducibility; equivalent sampling parameters could not be fixed in the web interface.

### Recruitment

We recruited a convenience sample of clinical nurse specialists (CNSs) via professional networks. Participants were presented with paired outputs in random order and blinded to allocation. Each participant evaluated the same eight preselected question-answer pairs, the maximum feasible within one hour based on pilot testing, using the Quality Analysis of Medical AI (QAMAI) tool (six domains rated on 5-point Likert scales) [8]. Readability was assessed with the Linguistic Features Toolkit (LFTK), including Flesch Reading Ease (FRE), Flesch Kincaid Grade Level (FKGL), and total words [9]. An LLM-judge, implemented with RAG GPT-4o, also rated all paired outputs using the QAMAI tool. Prompt templates and model configuration details, including API settings, are provided.

### Analysis

Two-tailed paired *t*-tests were used to compare Base and RAG scores when the within pair differences were approximately normally distributed; Wilcoxon signed-rank tests otherwise. Inter-rater agreement was assessed with intraclass correlation coefficients (ICCs 3,k); internal consistency with Cronbach α.

### Ethical Considerations

This study was approved by the King’s College London Minimal Risk Research Ethics Committee (MRSU 24/25 46882). Participants received a participant information sheet, provided written informed consent, and could withdraw at any time. Privacy and confidentiality were protected throughout. Participants received a £25 voucher for participation.

## Results

**Figure 1.**
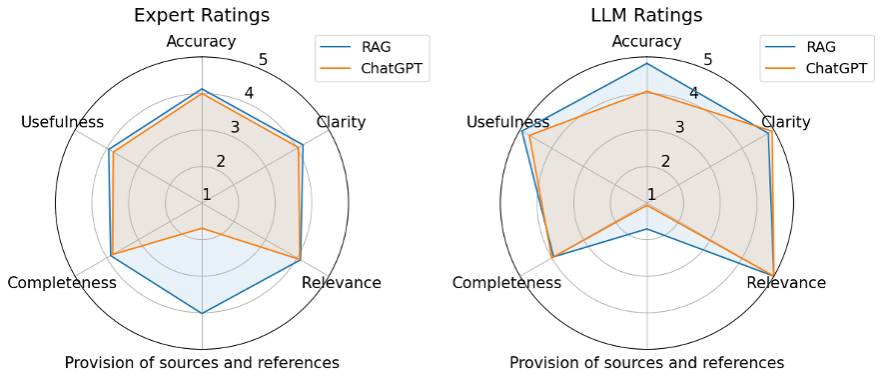
Mean QAMAI (Quality Analysis of Medical Artificial Intelligence) item-level domain scores (1 to 5, higher scores indicate higher quality) for RAG GPT-4o (RAG) and Base GPT-4o (ChatGPT). Left panel (Expert ratings): domain means calculated from 7 expert raters who each scored 8 matched answer pairs. Right panel (LLM ratings): domain means calculated from a single LLM-judge that scored all 17 matched answer pairs once. LLM: large language model; RAG: retrieval augmented generation.

Seven UK-based CNSs participated in the study (median post-registration experience 20 years; IQR 5‐30). Data collection ran from March-May 2025. Participants rated quality of RAG GPT-4o answers higher than those generated by base GPT-4o with highest mean difference in the domain, *Provision of Sources and References* (Table 1). Inter-rater agreement for total QAMAI scores was moderate (ICC 0.65, 95% CI 0.31-0.86); internal consistency was good (Cronbach α=0.81). The LLM-judge rated the quality of RAG GPT-4o answers higher than those generated by base GPT-4o (*P*<.001), again driven by the QAMAI *Provision of Sources and References* domain (*P*=.01) (Figure 1).

**Table 1.** Paired analysis of QAMAI^a^ and readability metrics.

QAMAI domain	RAG^b^ GPT-4o, mean (SD)	Base GPT-4o, mean (SD)	Mean difference (95% CI)	*P* value
Participants: 8 question-answer pairs evaluated				
Accuracy	4.14 (0.75)	3.98 (0.75)	0.16 (−0.05 to 0.37)	.11
Clarity	4.16 (0.65)	4.02 (0.77)	0.14 (−0.01 to 0.30)	.06
Relevance	4.11 (0.73)	4.09 (0.67)	0.02 (−0.26 to 0.23)	.78
Completeness	3.88 (0.90)	3.84 (0.93)	0.04 (−0.16 to 0.23)	.58
Provision of Sources & References	3.98 (0.94)	1.80 (1.24)	2.18 (0.92 to 3.44)	.03
Usefulness	3.96 (0.69)	3.80 (0.64)	0.16 (−0.10 to 0.42)	.17
Total (max=30)	24.23 (3.60)	21.54 (3.46)	2.69 (0.77 to 4.62)	.01^c^
LLM-Judge: 17 question-answer pairs evaluated				
Accuracy	4.82 (0.39)	4.06 (0.24)	0.76 (0.53 to 0.99)	<.001
Clarity	4.82 (0.39)	4.94 (0.24)	−0.12 (−0.35 to 0.11)	.16
Relevance	5.00 (0)	5.00 (0)	NA^g^	NA^g^
Completeness	3.94 (0.24)	4.00 (0)	0.06 (−0.18 to 0.06)	.32
Provision of Sources & References	1.71 (0.77)	1.06 (0.24)	0.65 (0.25 to 1.05)	.01
Usefulness	4.94 (0.24)	4.71 (0.47)	0.23 (−0.03 to 0.49)	.10
Total (max=30)	25.24 (1.35)	23.76 (0.56)	1.48 (0.76 to 2.20)	<.001^c^
LFTK^d^ Domain				
17 question-answer pairs evaluated				
FKGL^e^	7.2 (1.85)	6.2 (1.87)	1.00 (–0.1 to 2.2)	.08^c^
FRE^f^	67.9 (10.98)	75.2 (9.54)	−8.2 (–12.9 to –1.9)	.006^c^
Total words	370.8 (84.27)	308.5 (67.82)	62.3 (34.6 to 90.0)	<.001^c^

a QAMAI: Quality Analysis of Medical Artificial Intelligence.

b RAG: retrieval augmented generation.

c paired *t* test (otherwise Wilcoxon signed-rank)

d LFTK: linguistic features toolkit.

e FKGL: Flesch Kincaid Grade Level.

f FRE: Flesch Reading Ease.

g Not applicable.

RAG GPT-4o answers were longer (*P*<.001) and had a lower FRE score (*P*=.006), indicating more difficult-to-read text. FKGL did not differ significantly (Table 1).

## Discussion

Overall, experts and the LLM-judge rated both configurations highly for accuracy, clarity, relevance, and readability. RAG GPT-4o achieved higher overall QAMAI scores than Base GPT-4o, driven mainly by better provision of sources and references rather than perceived gains in accuracy or clarity. Although the LLM-judge identified differences in source attribution, it appeared to weigh provenance less than expert raters or had difficulty applying QAMAI in this domain, consistent with other studies [10]. RAG GPT-4o outputs were longer and less easy to read.

Although overall QAMAI scores were high, this does not eliminate the risk of clinically important failure in patient-facing cancer information. Errors may present as confident hallucinated claims, omission of safety critical details such as red flag symptoms or treatment risks, or overgeneralization that fails to address question intent. Provenance related problems are also possible, including outdated guidance and references that do not support the accompanying statements. Within this small sample, ratings did not suggest prominent safety related concerns, but larger evaluations are needed to characterize the frequency and impact of these types of error, and to determine whether high rubric scores and source attribution are sufficient safeguards for deployment without clinical oversight.

Improved provenance is expected with RAG, but the marginal benefit observed here must be weighed against the overhead of curating and maintaining a knowledge base, monitoring retrieval quality, and updating content as guidance changes. Although transparent sourcing may support perceived credibility, acceptability and trust in patient-facing information are also shaped by readability, tone, length, and cognitive load. Future work should evaluate whether optimized RAG configurations, and patient centered presentation formats improve usability, comprehension, acceptability, and perceived trust without increasing cognitive burden.

These findings suggest LLMs can generate patient-facing information that clinicians may judge as accurate, relevant, and readable, and could reduce the time required to draft materials. In this study, RAG improved overall QAMAI scores primarily through source attribution rather than perceived improvements in accuracy or clarity. Because RAG outputs were longer and less easy to read, any gains in transparency should be balanced against potential impacts on comprehensibility and health literacy.

Study limitations include a small convenience sample, expert ratings restricted to eight of 17 questions, use of a single LLM and UK knowledge base, and interface asymmetry between API and web access. Because equivalent sampling parameters could not be fixed in the web interface, this comparison should be interpreted as pragmatic and exploratory.

## Abbreviations

API: application programming interface
CNS: clinical nurse specialist
FKGL: Flesch Kincaid Grade Level
FRE: Flesch Reading Ease
ICC: intraclass correlation coefficient
LFTK: linguistic features toolkit
LLM: large language models
QAMAI: Quality Analysis of Medical Artificial Intelligence
RAG: retrieval augmented generation
